# Training muscle activation patterns of the lower paretic extremity using directional exertion improves mobility in persons with hemiparesis: a pilot study

**DOI:** 10.1186/s42490-021-00057-5

**Published:** 2021-10-29

**Authors:** Daniel Bourbonnais, René Pelletier, Joëlle Azar, Camille Sille, Michel Goyette

**Affiliations:** 1 School of Rehabilitation, Université de Montréal, P.O. Box 6128, Pavillon du Parc, Bureau 403-8, Station Centre-Ville, Montreal, QC H3C 3J7 Canada; 2 Centre for Interdisciplinary Research in Rehabilitation of Greater Montreal (CRIR), 300 Darlington Avenue, Montreal, QC H3S 2J4 Canada

**Keywords:** Dynamometer, Lower limb, Rehabilitation, Electromyography, Coordination, Single case study, Gait, Synergy

## Abstract

**Background:**

Controlled static exertion performed in the sagittal plane on a transducer attached to the foot requires coordinated moments of force of the lower extremity. Some exertions and plantarflexion recruit muscular activation patterns similar to synergies previously identified during gait. It is currently unknown if persons with hemiparesis following stroke demonstrate similar muscular patterns, and if force feedback training utilizing static exertion results in improved mobility in this population.

**Methods:**

Electromyographic (EMG) activity of eight muscles of the lower limb were recorded using surface electrodes in healthy participants (*n* = 10) and in persons with hemiparesis (*n* = 8) during an exertion exercise (task) performed in eight directions in the sagittal plane of the foot and a plantarflexion exercise performed at 20 and 40% maximum voluntary effort (MVE). Muscle activation patterns identified during these exertion exercises were compared between groups and to synergies reported in the literature during healthy gait using cosine similarities (CS). Functional mobility was assessed in four participants with hemiparesis using GAITRite® and the Timed Up and Go (TUG) test at each session before, during and after static force feedback training. Tau statistics were used to evaluate the effect on mobility before and after training. Measures of MVE and the accuracy of directional exertion were compared before and after training using ANOVAs. Spearman Rho correlations were also calculated between changes in these parameters and changes in mobility before and after the training.

**Results:**

Muscle activation patterns during directional exertion and plantarflexion were similar for both groups of participants (CS varying from 0.845 to 0.977). Muscular patterns for some of the directional and plantarflexion were also similar to synergies recruited during gait (CS varying from 0.847 to 0.951). Directional exertion training in hemiparetic subjects resulted in improvement in MVE (*p* < 0.040) and task performance accuracy (*p* < 0.001). Hemiparetic subjects also demonstrated significant improvements in gait velocity (*p* < 0.032) and in the TUG test (*p* < 0.022) following training. Improvements in certain directional efforts were correlated with changes in gait velocity (*p* = 0.001).

**Conclusion:**

Static force feedback training following stroke improves strength and coordination of the lower extremity while recruiting synergies reported during gait and is associated with improved mobility.

## Background

Stroke results in major limitations in terms of activities of daily living particularly with regard to mobility [1]. Approximately one-third of individuals who suffer a stroke have not regained independent mobility when discharged from rehabilitation and therefore are unable to walk unsupervised in the community [2]. This reduction in mobility leads to considerable secondary consequences, contributing to diminished social and valued roles [3].

People who experience a stroke demonstrate several sensorimotor impairments, including loss of muscle strength and impaired coordination that negatively impacts gait [4]. A recent meta-analysis found that progressive resistance exercise at higher intensities improves strength in this population [5]. However, systematic reviews and meta-analyses paint an unclear picture of the role that strength training of the lower extremity and associated strength gains play in improving gait in persons who have experienced a stroke [6–8]. Possible factors preventing strength gains from translating into functional performance may include suboptimal training intensities and duration and/or a lack of appropriate progression of the intervention [9, 10]. Alternatively, small effect sizes of strength training on gait parameters in this population may be the result of a lack of specificity of muscles selected and a failure to improve multiarticular movements requiring muscle activation of a coordinated group of muscles involved in locomotion. Strengthening exercises involving multiarticular muscles (e.g., leg press) appear to be better at improving strength and function in individuals with hemiparesis than isolated monoarticular exercises such as leg extensions [8].

Coordination of muscle groups involved in gait has been characterized in healthy subjects by EMG analysis of the lower extremity using a non-negative factorization technique [11]. This technique defines spatially grouped muscles (defined as synergies) and their corresponding temporal activation profile during the gait cycle. Four synergies (C1-C4) are usually sufficient to characterize gait in a healthy population [11, 12]. The C1 synergy consists of the activation of the Vastus Medialis (VM), Rectus Femoris (RF) and Gluteus Medius (GM) during the early stance phase; the C2 synergy, with activation of the Soleus (SOL) and Medial Gastrocnemius (MG), is related to forward propulsion during the terminal stance phase of gait; the C3 synergy consists of the activation of the Tibialis Anterior (TA) and RF observed during the initial swing phase while the C4 synergy with activation of the lateral (LH) and medial (MH) hamstring muscles is observed during the terminal swing phase. These four synergies contribute to important biomechanical functions that are required for normal non-impaired gait such as support, forward propulsion, mediolateral control and leg swing [13, 14].

The same or a reduced number of synergies revealed by factorization are observed in the paretic extremity of persons with hemiparesis following stroke [11, 12, 15, 16]. A reduced number of synergies is associated with impaired walking performance [17] and is explained by altered muscle activation or co-contractions of muscles involved in more than one synergy, effectively resulting in the merging of synergies [11, 12, 18]. Merged synergies during gait in people with hemiparesis typically involve synergies C1, C2 and C4, which are associated with different sub-cycles of gait [11–13].

People who demonstrate merged synergies following a stroke tend to exhibit a greater number of synergies after a training program for the upper or lower extremity [15, 19]. It has been suggested that a novel rehabilitation approach specifically aimed at retraining synergies may be required to address impairments in muscle activation and to improve function in persons with hemiparesis [13, 20]. During gait, the force that a foot exerted on the floor is opposed by the ground reaction force. The orientation of the ground reaction force (horizontal and vertical components) as well as the position of the joints (hip, knee, ankle) contribute to determine the activities of muscle during gait. Training the direction of force exertion of the extremity on a force plate may be considered an avenue for improving strength and coordination of the lower extremity and for practicing synergies observed during gait. First, directional exertion in healthy participants that requires individuals to control force feedback in two axes of the sagittal plane of the foot was found to recruit similar synergies to those reported in healthy participants during gait [21]. Secondly, persons who have experienced a stroke can coordinate ratios of increasing muscle activation and moments of force acting at different joints and produce a smooth exertion of force on a transducer localized under the foot [22]. Finally, modifying or training the exertion of moments of force and patterns of muscle activation can improve functional task performance [23, 24]. We therefore hypothesized that a progressive force feedback training program based on controlled directional exertions on a static dynamometer, requiring controlled moments of force at various joints of the lower extremity, would recruit synergies identified during gait and that such a program could be used to improve mobility in participants with hemiparesis.

The first objective of this study was to determine if directional exertions and static plantarflexion resulted in similar muscle activation patterns in people with hemiparesis and healthy participants, and if such activation patterns were similar to synergies reported during gait in healthy persons [11]. The second objective was to determine if a training program based on static directional exertions of the lower paretic extremity and static plantarflexion resulted in improved mobility and in people with hemiparesis. We also verified if changes in Maximal Voluntary Effort (MVE), in the accuracy required to perform the exertion exercise, and in muscle activation patterns are observed and associated with improved mobility following training.

## Methods

### Participants

The control group consisted of a sample of ten healthy participants (5 males; 5 females) aged between 18 and 30 (25.3 ± 3.1) years with no reported neurological conditions or musculoskeletal impairments that could limit their mobility. The experimental group included eight participants (6 males; 2 females) aged between 48 and 72 (56.0 ± 8.5) years with hemiparesis as a result of stroke affecting their dominant (*n* = 4) or nondominant (n = 4) side. The participants in the experimental group met the following inclusion criteria: (1) medically stable, (2) no reported cognitive disorders, aphasia or visual deficits precluding the performance of tasks, (3) ability to walk 10 m uninterrupted with or without orthoses or mobility aids, (4) no reported pain associated with musculoskeletal problems of the trunk and/or lower extremity, and (5) resided within 20 km of the laboratory. The first part of the study involved all healthy and hemiparetic participants. However, only four participants with hemiparesis (subjects 5, 6, 7 and 8) participated in the second part of the study involving the training protocol. Ethical approval was obtained from the Research Ethics Committee of the Centre for Interdisciplinary Research in Rehabilitation of Greater Montreal (CRIR) (1296–0218 and 1344–0518). Prior to their participation, all participants received detailed information about the study and the nature of their participation, and provided verbal and written consent.

The basic demographic and clinical characteristics of the participants with hemiparesis are presented in Table 1. Motor function of the paretic lower limb was evaluated using the Impairment Inventory component of the Chedoke-McMaster Stroke Assessment quantifying the stage of recovery of the leg and foot with a maximal score of 7 (higher scores indicating less impairment) [25]. Muscle tone of the triceps surae was evaluated based on the Modified Ashworth Scale [26], with a score of 0 indicating no resistance and a score of 4 marked spasticity. Gait velocity was assessed using GAITRite® (CIR Technologies, Franklin, N.J.) with participants using their walking aid or ankle foot orthosis. The study was conducted in the Pathokinesiology Laboratory located within the Centre for Interdisciplinary Research in Rehabilitation of Greater Montreal (CRIR) of the Integrated Health and Social Services University Network.

**Table 1:** Clinical characteristics of the stroke participants

Subject	Age (year)	Gender	Affected side	Chedoke McMaster Leg (7)	Chedoke McMaster Foot (7)	Modified Ashworht Scale (4)	Walking aid	Gait velocity (m/s)
**1**	58	M	L	6	3	1	AFO / WS	0.83
**2**	52	M	R	4	4	0	AC	0.88
**3**	49	F	R	5	5	0	None	1.42
**4**	48	F	R	5	4	1	None	1.30
**5**	50	M	L	7	6	0	QC	1.03
**6**	58	M	L	7	6	3	None	0.86
**7**	59	M	L	5	3	3	QC/AFO	0.11
**8**	74\	M	R	6	2	2	AFO / AJ	0.73

*AFO* Ankle foot orthosis

*WS* Walking stick

*AC* Adjustable cane

*QC* Quadpod cane

### Dynamometry

**Fig. 1:**
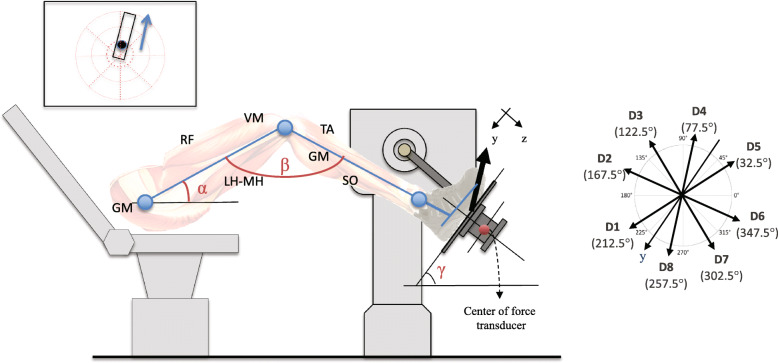
Experimental set-up. The participant’s foot is firmly attached to a force transducer interfaced with a laboratory computer. The forces exerted in the Y and Z axes are displayed as a cursor on a screen located beside the participant. The participant’s task is to exert progressive static exertion by moving the cursor within a corridor in eight successive directions. Plantarflexion is performed and trained using a unidirectional cursor displaced in proportion to the moment of force measured in the X axis

In this study, participants were required to exert static exertion in different directions in the sagittal plane using the lower extremity. The apparatus used in this study has been previously described [21]. The participants were seated semi-reclined on an adjustable chair (Biodex Medical Systems, NewYork, USA) with either the non-dominant foot (healthy participants) or the paretic foot (hemiparetic participants) secured on the force platform with large Velcro straps (Fig. 1). Participants (Table 1; subjects 1, 7 and 8) who required an ankle foot orthosis for gait did not use their orthosis while performing the directional exertion exercises. The height and position of the chair were adjusted to ensure that the foot was positioned horizontally at a 55 degree angle with 20 degrees of hip flexion and 125 degrees of knee flexion (Fig. 1). The kinetic output from the force platform (AMTI model MC3–1000, Advanced Manufacturing Technology Inc., Massachusetts, USA) was digitized using an acquisition card and recorded at a frequency of 100 Hz. This experimental set-up allowed for the measurement of vertical and horizontal forces (Fy and Fz) and moments of force (Mx) exerted under the foot in the center of the force transducer [21]. A force feedback cursor was displayed on a screen placed beside the participant for viewing. The cursor moved horizontally or vertically in proportion to the Fz and Fy force exerted in the center of pressure of the foot for directional exertions and vertically only for plantarflexion in proportion to the Mx. Once seated and securely attached to the apparatus, participants were given time to familiarize themselves with the task and were then asked to progressively move the cursor within a corridor displayed on a monitor oriented in one of eight specific directions (Direction 1 to Direction 8) in order to perform maximal exertion (Fig. 1).

### Surface EMG recordings

Surface EMG of the TA, SO, MG, VM, RF, LH, MH and GM muscles were recorded on the left (non-dominant) lower extremity of healthy participants and on the paretic side of hemiparetic participants and recorded at a frequency of 1200 Hz. These recordings were performed during a single session among all healthy participants (part 1 of the study) and the first four stroke participants (part 1 of the study) and during repeated sessions at different timepoints in the study for the last four hemiparetic subjects (part 1 and 2 of the study): at baseline (E1, E4 and E7), upon withdrawal (E8, E10 and E13) and at follow-up (E14). The recording sites for each muscle were determined based on SENIAM recommendations [27]. These sites were shaved and cleaned with alcohol [28] and surface electrodes (Ambu BlueSensor M) were positioned perpendicular to the muscle fiber orientation of each muscle at a 1 cm inter-electrode distance. EMG signal recordings were verified visually during contractions performed against gravity or manual resistance.

### Data processing

EMG signals were recorded and filtered using a fourth-order Butterworth zero-lag bandpass filter with cut-off frequencies set at 10 and 400 Hz. Subsequently, EMG values were root-mean- squared (RMS) with a centered 250 ms moving window to generate linear envelopes. Kinetic and EMG data were collected while the participant exerted between 80 and 90% of the directional exertion. Three cycles were retained for analysis based on minimal EMG RMS variation coefficients.

### Part 1 of the study: comparing muscle activation patterns during directional exertion and plantarflexion between groups and with synergies previously reported during gait

Participants were asked to perform a progressive MVE by moving a cursor within a corridor displayed on a monitor for each of the eight directions (Fig. 1). Once the maximal efforts completed, the healthy participants sequentially performed the exertion task at 20 and 40% of their MVE in each of the eight directions using a target indicated within the corridor and maintain the exertion for 2 s (Fig. 1). A one-minute rest period was allocated between each direction. Since participants who had experienced a stroke required more time to perform the exercises, only one level of force was tested to minimize fatigue. Four stroke participants were evaluated at 20% of their MVE and the last four stroke participants (Table 1) at 40% of their MVE in order to cover the range of exertion utilized with the healthy participants. Maximal voluntary plantarflexion was not evaluated given that the healthy participants could exceed the maximal capacity of the transducer of 110 Nm [29]. The maximal plantar flexor torque of a group of hemiparetic participants with similar age and gender distribution is approximately 20 Nm [30]. Both healthy and hemiparetic participants were therefore instructed to perform 40% of this estimated maximal plantar flexor torque guided by visual feedback.

The RMS of EMG values was calculated for each individual for all directions of exertion and plantarflexion and the peak RMS value was determined. The RMS of EMG values was amplitude normalized from its peak value at each session and expressed between 0 to 1 to reduce inter- and intra-subject variability. These normalized EMG values calculated for each muscle did not differ between levels of exertion in healthy participants (within group: 20 and 40%, *p* < 0,050) and the EMG data was pooled. Patterns of muscle activation consisting of the averaged muscle activation of the eight muscles in a given direction or during plantarflexion were compared between the healthy and hemiparetic participants using cosine similarities (CS) [31]. CS were calculated as the inner product of two muscle vectors representing the averaged muscle activation of the eight muscles in a given direction or plantarflexion (Fig. 3). As previous studies, CS value above 0.80 was used as a criterion to define similarity between a pair of muscle vectors [32, 33].

CS were also used to compare the averaged activation patterns of the eight muscles for each direction of exertion with averaged values of the previously reported synergies extracted using non-negative matrix factorization of the same eight muscles during healthy gait [11, 15]. The CS were calculated using the averaged muscle activation in a given direction or during plantarflexion, and the previously reported synergies (C1, C2, C3, C4) during gait [11, 15] for both groups of participants. The synergies during gait were therefore not obtained from our participants but rather estimated from data reported in the literature of the same muscle groups recorded in a cohort of healthy subjects of similar age.

### Part 2 of the study: training program using directional exertion of the lower limb

#### Design

**Fig. 2:**
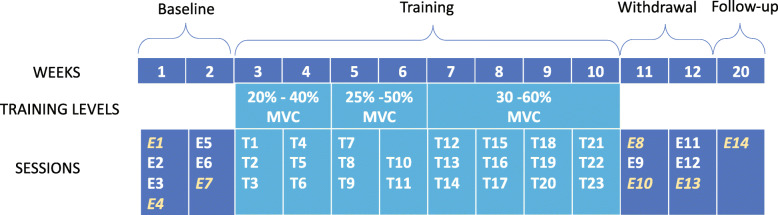
Training program and study design. Four stroke persons participated in a training program based on directional and plantarflexion static exertions. Mobility parameters (gait velocity and Timed Up and Go test results) were evaluated at every session at baseline (E1 to E7), during the treatment (T1 to T23) and upon withdrawal (E8 to E13) and one other time 2 months later (E14). Muscle activity patterns were evaluated during static directional exertions in sessions identified in yellow and italics at baseline (E1, E4, E7), upon withdrawal (E8, E10, E13) and at follow-up (E14). During the intervention period, MVEs were measured at the beginning of each week. Participants were asked to perform eight controlled static exertions in the eight directions and plantarflexion exertion during the first session and twelve during the second and third sessions of each week at an increasing level of exertion each week

A series of four single-case studies using a single standardized protocol was completed. The protocol consisted of 37 sessions distributed across 4 periods (baseline, training, withdrawal, and follow-up) (Fig. 2). The baseline period consisted of 7 sessions (4 measurements during week one and 3 during week two). The training program comprised 3 sessions of training per week for 8 weeks (one session was not provided due to a statutory holiday). The training period was followed by a withdrawal period consisting of 6 sessions without training and a single follow-up session, 8 weeks after the withdrawal period.

During training, the subjects were instructed to perform maximal exertion exercises in eight successive directions while remaining within the corridor displayed on the monitor as much as possible. During the training period, the maximal exertion produced by the subjects was measured at the first session of every week and used to re-scale the requested directional exertion tasks while the maximal value of plantarflexion remained the same throughout the training program. During the first session of a given week of training, 8 repetitions were requested and 12 repetitions for the second and third sessions of the week. Two levels of exertion (i.e., directional exertion) were used during the training period and were increased every 2 weeks (20 and 40% weeks 1–2, 25–50% weeks 3–4, and 30 and 60% weeks 5–8). However, the levels of exertion remained constant for the plantarflexion exercise.

### Clinical assessments of mobility

At the beginning of each of the 37 sessions, gait velocity was measured using GAITRite® and mobility and balance using the Timed Up and Go (TUG) test. The research assistant utilized standardized protocols for each measure including verbal instructions provided. Participants were blind to scores from previous sessions. Measures were taken in the same order and the same shoes/ orthoses/walking aid were worn for each evaluation. Participants with hemiparesis were asked to walk three times on the GAITRite® system at their natural speed like they were “going to their kitchen” and the gait parameters of the three trials were averaged for analysis. The TUG test was performed 3 times with a digital stopwatch and the mean value was retained for analysis. Both the GAITRite® system [34] and the TUG test [30] have demonstrated good test-retest reliability.

Gait speed and TUG measurements for each participant were plotted on a graph at each session and initially analyzed visually to identify trends in the progression of mobility performance before, during and after the training period. Non-parametric Tau-U statistics were used to determine if there was a significant change between the baseline and withdrawal phases of the intervention [35]. The Tau-U statistic represents the percentage of data that is modified over time after controlling for the trend in baseline. The index varies between 1 and − 1. For example, a value of 0.80 or − 0.80 indicates that 80% of the data either increased or decreased between phases, respectively. The statistic was calculated using a free Web-based application [36].

### Comparing force production and accuracy of exertion before and after training

MVE values recorded for each direction and during plantarflexion across the various sessions during the baseline (E1, E4, E7), withdrawal (E8, E10, E13) and follow-up (E14) periods were averaged. Similarly, an accuracy index was calculated (i.e., the mean of the RMS values for the distance between the vector and the center of the corridor calculated between 80 to 90% of the progressive exertion. For MVE and accuracy analysis, the pre-training values (E1-E4-E7) and post-training vales (E8-E10-E13) were compared using a two within-subjects factor (Period, Direction) repeated-measures ANOVA. When there was significant interaction (Period*Direction), paired t-tests were performed between the pre- and post-training values for each of the 8 directions of exertion. To account for multiple comparisons, the Benjamini-Hochberg false discovery rate correction was used to determine statistical significance. Spearman rho correlations between pre- and post-intervention delta scores for MVE, accuracy, gait velocity and TUG values were performed.

### Comparing patterns of muscle activation before and after training

The normalized EMG activity patterns of the eight muscles were averaged for each of the 4 participants with hemiparesis and for each direction during the baseline (E1, E4 and E7) and withdrawal (E8, E10 and E13) phases of the intervention. The CS comparing these patterns at baseline and upon withdrawal was calculated. Similarly, normalized muscle activities during the same periods were averaged for the plantarflexion condition and the CS was calculated. The patterns were considered similar when the CS was higher than 0.80 [32, 33].

## Results

### Part 1 of the study: similarities between muscle patterns during directional exertion and plantarflexion and synergies reported during gait

**Fig. 3:**
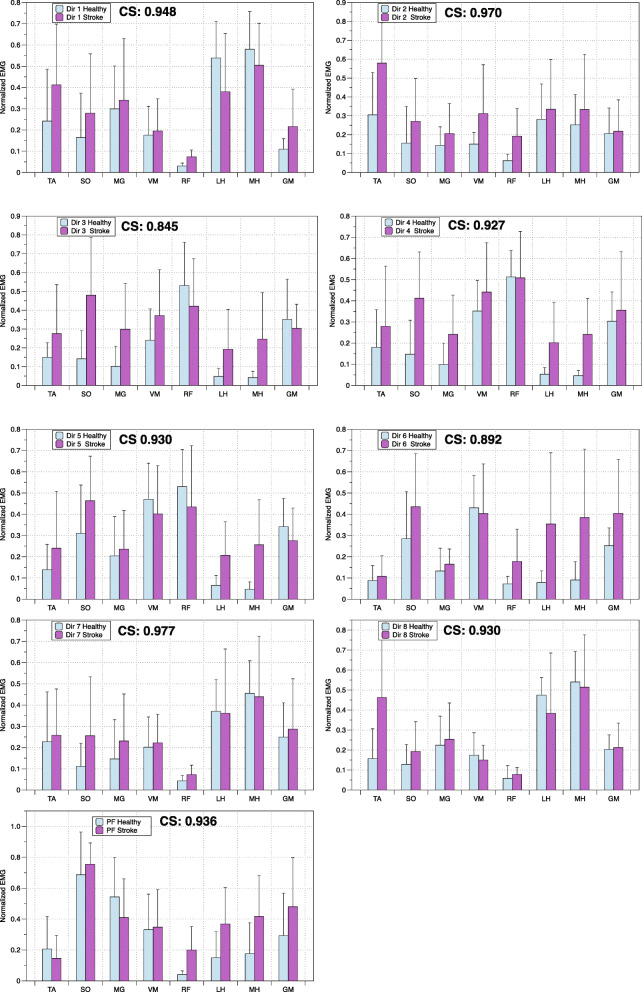
Comparing patterns of muscle activation in healthy and stroke participants. Cosine similarities (CS) comparing the patterns of normalized EMG muscle activity recorded in healthy and stroke subjects for each direction (Dir 1- Dir 8) and plantarflexion (PF)

The normalized EMG activity patterns of muscles across the eight directions for both the healthy participants (*n* = 10) and the stroke participants (*n* = 8) are illustrated in Fig. 3. The CS varied between 0.845 to 0.977. Lower values were observed in directions 3 and 6, demonstrating increased activity in the LH and MH in stroke participants as compared to healthy participants. In addition, increased activity of the SO was observed in stroke participants in direction 3. Overall, the results indicate that the normalized muscle activity patterns were similar for both groups across the 8 different directions.

**Fig. 4:**
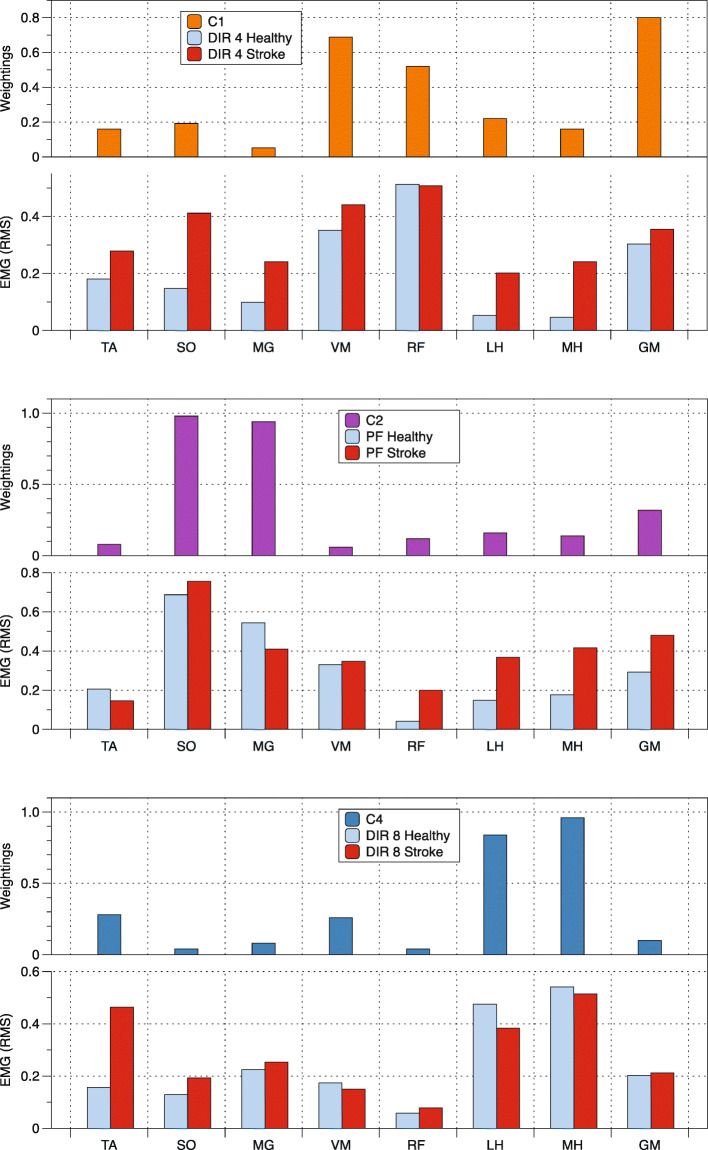
Comparing patterns of muscle activation with synergies reported during gait. Averaged muscle activity in the healthy and stroke group during specific directional efforts (D2, D4 and D8) and plantarflexion (PF) are compared to averaged synergies during gait in healthy subjects as reported in the literature [11, 15]

Muscle activation patterns during directional exertion were compared to the averaged synergies extracted for the same muscles during healthy gait [11, 15] (see Table 2). For both the healthy and hemiparetic groups, high CS values between a synergy documented during gait and muscle activation patterns reported during static exertions are respectively: C1 and Direction 4; C2 and plantarflexion and C4 and Direction 8 (Table 2). In general, the CS calculated for participants with hemiparesis were lower than those in the healthy group. Muscle activation during directional exertions and the synergies previously reported during gait [11, 15] that demonstrated greater highest CS values, above 0.80 in Table 2, are illustrated in Fig. 4.

**Table 2:** Cosine similarities (CS) values between EMG activities during the directional exertions (D1 to D8) and plantarflexion (PF) and the synergies reported in the literature during gait for both the healthy and stroke groups. Bold values indicate the highest cosine similarity index for each muscle synergy

	Synergy	Direction of exertion								
Group		D1	D2	D3	D4	D5	D6	D7	D8	PF
Healthy	C1	0.460	0.670	0.895	**0.912**	0.904	0.851	0.643	0.553	0.581
	C2	0.520	0.564	0.440	0.425	0.577	0.634	0.467	0.497	**0.935**
	C3	0.377	0.650	0.757	**0.771**	0.674	0.435	0.466	0.362	0.382
	C4	0.947	0.814	0.264	0.299	0.291	0.414	0.927	**0.951**	0.392
Stroke	C1	0.568	0.676	0.812	**0.875**	0.828	0.824	0.678	0.547	0.714
	C2	0.646	0.546	0.748	0.653	0.704	0.665	0.632	0.538	**0.847**
	C3	0.563	**0.778**	0.686	0.723	0.677	0.444	0.505	0.605	0.414
	C4	0.835	0.728	0.506	0.516	0.542	0.709	0.851	**0.865**	0.593

### Part 2: clinical training

All 4 subjects with hemiparesis completed the treatment program and evaluation sessions. In one subject (subject 7) a two-week interruption was required after E4 due to the flu. A technical problem led to one missing measure of velocity that was rapidly resolved (sessions 9, 15, 16 for subjects 5, 6 and 7 respectively).

### Clinical measurement of mobility during training

**Fig. 5:**
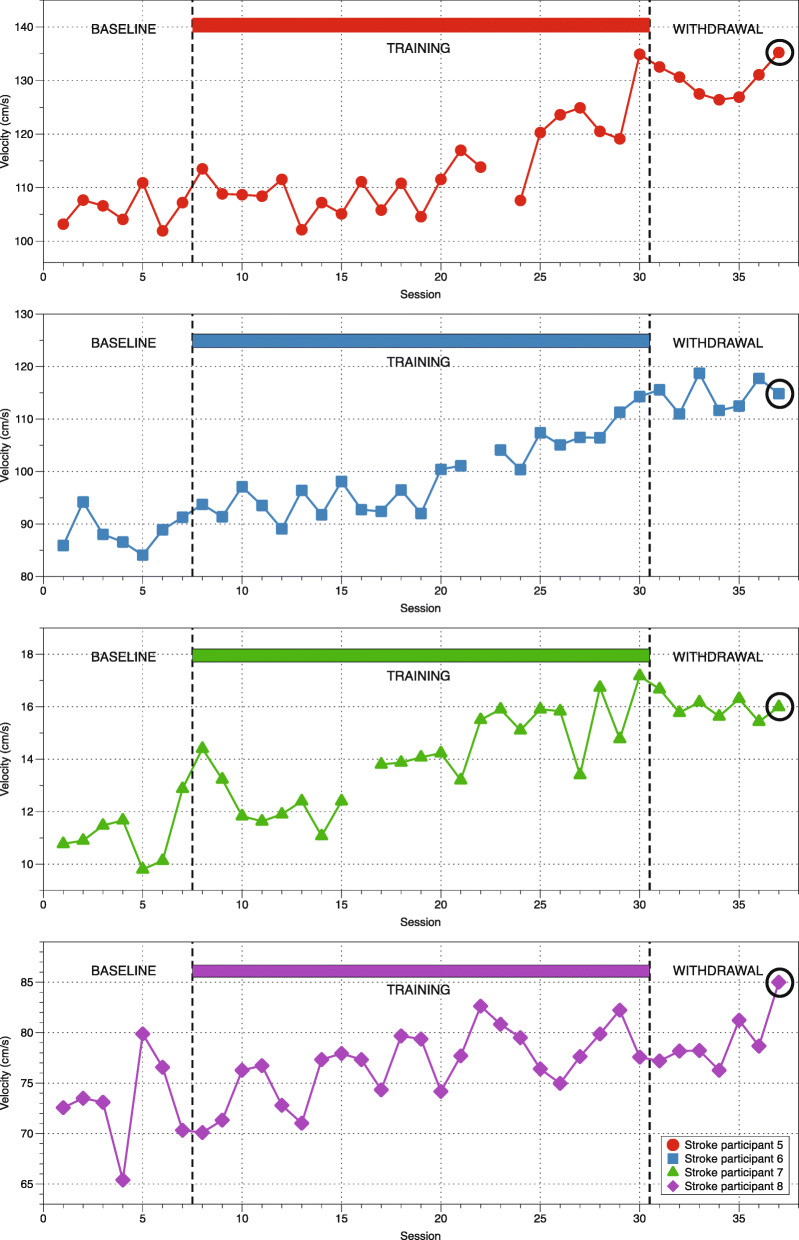
Gait velocity performance during training. Three averaged measurements of gait velocity at different sessions during baseline, training, withdrawal and follow-up (indicated by a circle) for the four stroke participants

**Fig. 6:**
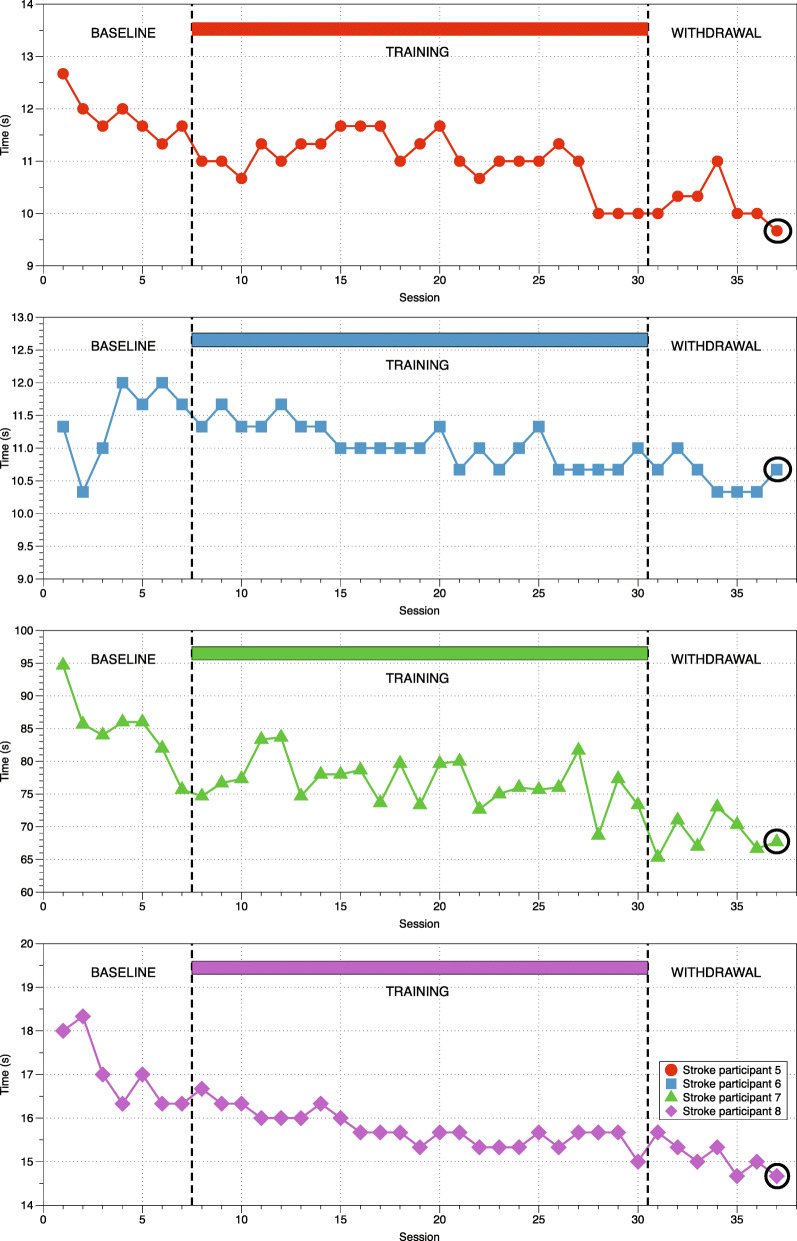
Timed Up and Go performance during training. Two averaged TUG measurements at different sessions during baseline, training, withdrawal and follow-up (indicated by a circle) for the four stroke participants

All subjects achieved an increase in gait velocity throughout the training period that was maintained during the withdrawal and follow-up period (Fig. 5). Tau analysis indicate that the improvements in gait velocity were significant for all subjects (*p* < 0.032) when baseline and withdrawal periods are compared. Mean improvements vary among subjects and were 22, 30, 44 and 7% of the initial scores, respectively. Improvement in TUG scores between baseline and withdrawal was also significant (*p* < 0.022) with a decrease of 13, 8, 19 and 11% of the time taken to complete the test (Fig. 6).

### Force production and accuracy of exertion before and after training

**Fig. 7:**
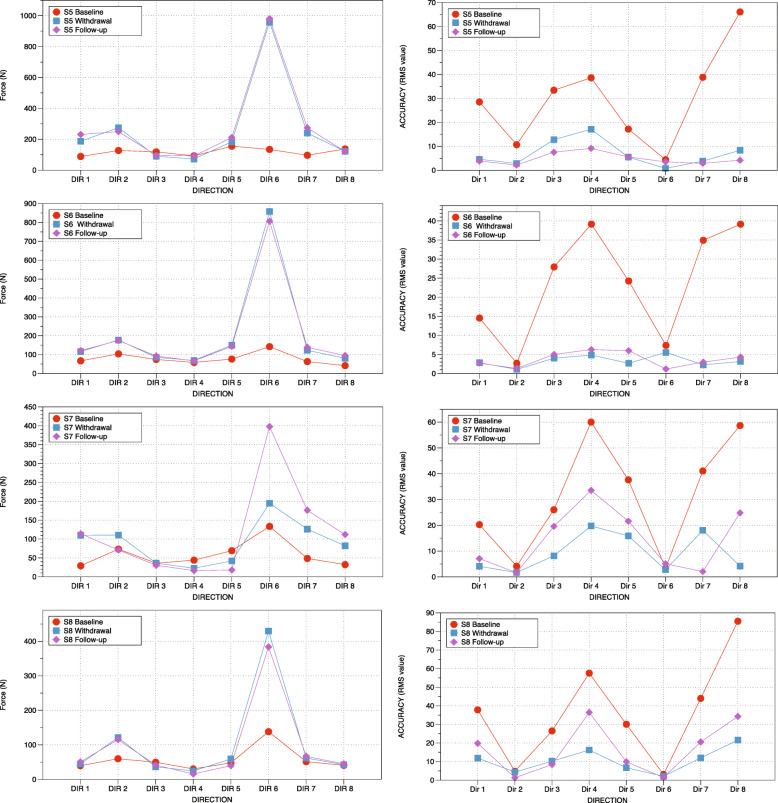
Improved maximal voluntarily effort and accuracy of exertion during training. Averaged MVEs and precision indexes in the four stroke participants to training according to directional exertions at baseline, during withdrawal and follow-up

Changes in MVE and accuracy indexes at baseline, withdrawal and follow-up period are illustrated according to the directions of exertion (Fig. 7). MVE post-intervention (166.5 N ± 42.2) values were greater than pre-intervention values (55.3 N ± 10.1) with a significant main effect between periods (F = 11.60, df = 1,21, *P* < 0.040, n^2^ = 0.795). There was also a main effect between directions of exertion. As would be expected, some directions had greater MVE than others (F = 15.75, df = 7, 21, *p* < 0.001, n^2^ = 0.840). There was also a significant interaction effect (Period*Direction) (F = 5.88, df = 7, 21, *p* < 0.001, n^2^ = 0.662), suggesting that the period had different effects on MVE for the 8 directional exertions performed. However, post-hoc comparisons were not statistically significant for any of the eight directional exertions between pre- and post-training after correcting for multiple comparisons.

Analysis of the accuracy of the directional exertions determined that there was a significant main effect for the baseline and withdrawal periods. RMS values were lower upon withdrawal than at baseline (F = 457.96, df = 1, 21, *p* < 0.001, n^2^ = 0.993), indicating improvement in performing the exertion within the required corridor (Fig. 1). As would be expected, there was also a main effect between the different directions of exertions (F = 14.85, df = 7, 21, p < 0.001, 0,832). There was also a significant interaction effect (Period*Direction) (F = 25.02, df = 7, 21, p < 0.001 n^2^ = 0.893), which suggests that the period had different effects on accuracy for some of the 8 directional exertions performed. Post-hoc comparisons revealed that accuracy improved in directions 3, 4, 5, 7 and 8 post-training vs pre-training (all *p* < 0.006) after correcting for multiple comparisons.

Delta scores for MVE had a positive correlation with delta scores for gait velocity in directions 2 and 6 (rho = 1.00, *p* = 0.000). Delta scores for accuracy had a positive correlation with delta scores for gait velocity in directions 6 and 7 (rho = 1.00, p = 0.000). Delta values for MVE and accuracy had no significant correlations with TUG values.

### Muscle activation patterns before and after training

**Fig. 8:**
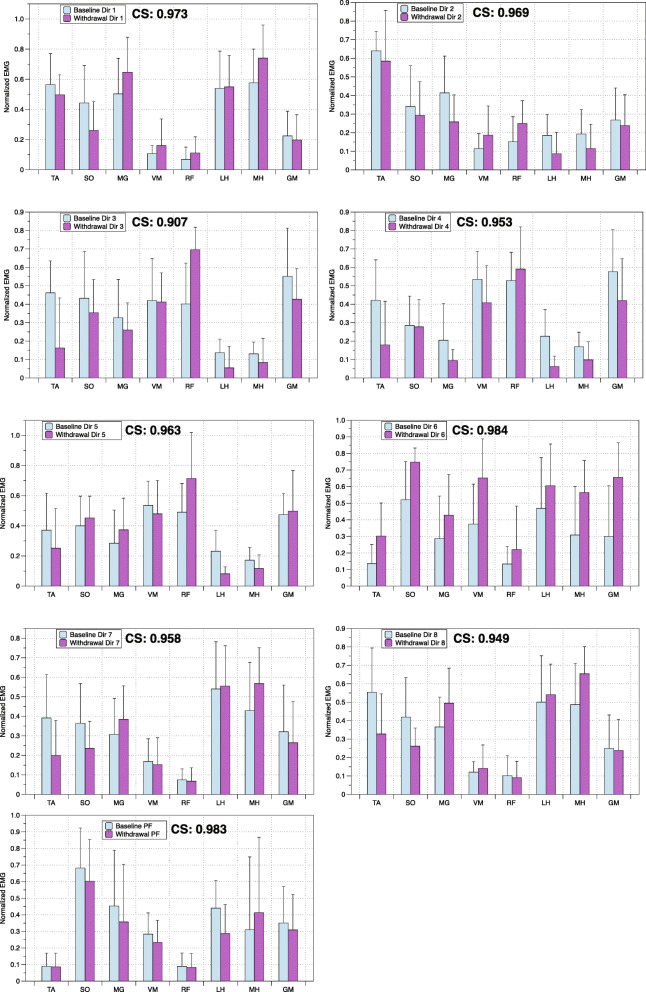
Muscle activation patterns before and after training. Cosine similarities comparing the patterns of normalized EMG muscle activity recorded in stroke subjects for each direction and plantarflexion during the baseline and withdrawal periods of training

The normalized EMG activity values for the eight muscles were averaged for each participant with hemiparesis during the baseline (E1, E4 and E7) and withdrawal (E8, E10 and E13) phases of the intervention (Fig. 8). CS comparing muscle patterns at baseline and upon withdrawal showed high values for each direction of exertion, ranging between 0.907 to 0.984. Similarly, the normalized muscle activity values during the baseline and withdrawal periods were averaged for the plantarflexion condition. CS also showed a high value of 0.983. This suggests that the muscle activation patterns during directional exertion and plantarflexion were comparable following training.

## Discussion

Muscle activation patterns were similar during static exertions performed at the foot in both groups of participants. As hypothesized, plantarflexion and some of the directional exertions (direction 4 and 8) demonstrated muscle activation patterns consistent with synergies reported during healthy gait. The force-feedback training based on directional exertions resulted in significant improvement in clinical mobility (gait speed and time to perform a functional task such as standing and walking) in people with hemiparesis. Most importantly, improved performance during directional exertion before and after the training period (i.e., strength and accuracy) was observed. It is suggested that the increased efficiency in the ability to control torque exerted at different joints of the lower extremity and the opportunity to practice muscle activation patterns similar to synergies normally recruited during gait contributed to the improvement in mobility.

### Muscle activation pattern similarities during directional exertions and gait

Mean muscle activation patterns observed during directional exertions and plantarflexion were similar between groups (Fig. 3), although muscular patterns in some directions and during plantarflexion in both groups were similar to synergies that have been identified in healthy gait (Fig. 4) [11, 15], CS values were lower in hemiparetic subjects (Table 2). Impaired levels of muscle activation or increased co-contraction of antagonist muscles in the paretic limb (which is well documented in this population) is the most probable explanation for these findings [37]. For example, mean paretic muscle activity during exertion in direction 4 is similar to synergy C1 (Fig. 4). However, activity of the SO, LH and MH is greater in hemiparetic participants than healthy participants. Similarly, mean activation values for LH, MH and GM during plantarflexion are greater in participants with hemiparesis compared to healthy participants and demonstrate less similarity to the C2 synergy. Lastly, mean activity of the TA muscle in hemiparetic participants is elevated compared to the control group during exertion in direction 8, with a difference in activation of this muscle in the C4 synergy. Although co-activation of a few muscles is observed during these directional exertions, the muscle activation patterns of hemiparetic participants as evaluated using CS are similar to those of healthy subjects.

### Training based on directional exertions and plantarflexion improves gait velocity and TUG test results

Although there were a considerable number of experimental and training sessions, subject compliance to the protocol was high. Training resulted in improved gait velocity in the four subjects (Fig. 5). Analysis of the gait-related spatiotemporal parameters indicates that the common strategy used by all subjects to improve gait velocity was to increase the step length of the paretic side and decrease the percentage of double leg support during a gait cycle. Subjects 5, 6 and 7 also increased their cadence. These observations still need to be validated in a larger population but suggest that various strategies may be used to improve gait velocity.

There was less improvement in the amount time required to perform the TUG test (Fig. 6), a functional task of standing and walking. This is not unexpected as performance of the TUG involves aspects other than walking.

### Training improves strength and accuracy during directional exertions

The training program was designed to require coordinated moments of force acting at different joints of the lower extremity while progressively increasing the level of force required. The MVE was increased following the training but the significant interaction between the direction and period indicates that the MVE was not increased similarly over the 8 directional exertions performed. As depicted in Fig. 7, an increase in strength was particularly apparent in directions 2 and 6 for the four subjects with hemiparesis. In these directions, the action of the force exerted at the foot is close to the center of rotation of the joints of the ankle, knee and hip, consequently generating less moments of force at these joints [21]. Unsurprisingly, all hemiparetic and healthy participants reported that the exertions were easier to produce in these directions. Exertions in these directions require concurrent activation of flexor (direction 2: TA, LH, MH; GM) or extensor (direction 6; SO, VM, GM) muscles acting at different joints of the lower extremity corresponding to gross movements observed with early motor recovery of the lower extremity in persons with stroke [38]. Overall, the increase in strength was more apparent in directions 2 and 6 as it was easier to coordinate the muscle groups involved to produce the forces.

It was expected that training would improve not only strength but also accuracy of the directional exertions. Improved accuracy was observed in directions requiring the production of larger combinations of torque at different joints, including hip flexion, knee extension, dorsiflexion of the ankle (Direction 3, 4, 5), hip extension and knee flexion with ankle plantarflexion (direction 7 and 8) [21]. Subjects reported that exertions in these directions were more difficult to perform and control. Nonetheless, improvement in accuracy suggests that stroke subjects improved the coordination of moments of force exerted at the different joints of the lower extremity during static exertions.

In summary, it is possible that improvements in strength and accuracy, which are thought to reflect improved coordination of torque produced at different joints, contributed to the improvement in mobility. Significant relationships between improvement in MVE and accuracy in task performance in some directions with changes in gait velocity following training support this hypothesis. Alternatively, averaged muscle activation patterns before and after training were similar as indicated by the high CS values for the different directions of exertion and plantarflexion (Fig. 8). It appears that improved performance to increase and coordinate the moments of force were not paralleled with the changes in muscle activation patterns during directional exertions and plantarflexion. It is also possible that the electromyographic recordings or CS were not sensitive enough to detect a difference in the muscle activation patterns before and after the intervention or that improved coordination resulted from a temporal parameter that was not measured. Although the CS comparing muscle patterns at baseline and upon withdrawal were similar for each direction of exertion and plantarflexion, it remains possible that the training, which consisted of improving strength and coordination of muscles of the lower extremity, had a carry-over effect to gait. In other words, although the patterns of activation during dynamometric efforts did not change before and after treatment, it is possible that the synergies during gait were nonetheless modified after training. Analysis of the synergies during gait before and after the training program would be required to answer this question*.*

### Synergies reported during healthy gait are recruited during training

Improvements in strength and accuracy in some directions of exertion were achieved using muscle activation patterns similar to synergies observed during healthy gait. For example, accuracy greatly improved during exertions in directions 4 and 8 (Fig. 7) for which muscle activation patterns were found to be similar to the synergies C1 and C4, respectively. Previous findings indicated that synergies C1 and C2 and synergies C1 and C4 were the two more commonly merged synergies in hemiparetic subjects [11, 13]. Moreover, when synergy C2 is merged with another synergy, the person with hemiparesis demonstrates a decrease in balance control and gait patterns, indicating that an independent (“not merged”) synergy is critical to walking and balance performance [39]. Training these different synergies through directional exertions and plantarflexion therefore provided an opportunity to practice and voluntarily recruit these synergies. It is possible that these muscle patterns were not initially used by hemiparetic participants before the training but were slowly integrated into the gait pattern during the training. Training may have contributed to an improvement in gait velocity by decreasing muscle co-contraction and dissociating merged synergies [11, 15]. Analysis of synergies during gait before and after the training program would need to be verified if training decreases co-contractions and modifies the activation profiles of the synergies. Previous research suggests that locomotor rehabilitation has the potential to influence not only the synergy composition but also the timing, which can lead to improvements in gait [15]. Accordingly, it remains possible that the force-feedback training improved the control of the synergies recruited during gait and contributed to the improvement in mobility in the stroke subjects.

### Limitations

Our results indicate that some of the averaged patterns of muscle activation during static efforts are similar to synergies reported during sub-cycles of gait in healthy subjects. A major limitation of our study is that the synergies during gait were not recorded in our participants but rather estimated from data of healthy subjects previously reported in the literature. A more rigorous approach would be to compare the muscle activation patterns during static efforts to synergies obtained in the same participants during gait and this for a larger group of participants. Moreover, as previously mentioned the synergies could be evaluated in participants having experienced a stroke before and after the intervention to determine if changes in the number or merging of synergies occur as observed in other rehabilitation interventions [15, 19].

Comparisons of muscle activation patterns and synergies were performed using cosine similarities. This metric is a non centered correlation and is frequently used in the literature to compare synergies with a pre-established level of usually above 0.80 [32, 33]. However, the accuracy of this metric to determine what changes are meaningful and its sensitivity to find differences between conditions and time periods remains to be established.

The results of the present experiment utilizing a multiple baseline design in a small sample suggests proof of concept of the instrument and intervention. Having only a sample size of four participants impacts generalizing the results to a larger population*.* There is no randomization or replication of the baseline or intervention phases in these single-cases series. Therefore, this design lacks internal validity and generalizability of results.

It remains to be established how the training could implemented in a clinical setting considering the instrumentation required. The training could be enhanced by verifying that the directions of exertion chosen are optimal for improving the combination of moments of force or patterns of muscle activation resembling the synergies reported during gait (Fig. 4). It is possible that some hemiparetic participants used different locations for the center of pressure (COP) while performing the directional exertion or had difficulty stabilizing the COP, modifying the synergies of muscles acting at the ankle. Feedback on the location of the COP exerted by the foot may also be considered for future studies as well as the use of a transducer with a higher tolerance in order for the level of torque required for plantarflexion during training to be increased.

## Conclusion

Directional exertion training directional with force feedback on a static platform attached to the foot can be used to improve the accuracy and level of moments of force produced at various joints of the lower extremity in hemiparetic subjects and results in improved gait velocity. These findings suggest that further exploration of the training protocol involving static exertions with force feedback to improve mobility in this neurological population with a larger group of participants allowing a more robust design is warranted.

## Data Availability

Please contact the corresponding author for data requests.

## Abbreviations

CRIR: Centre for Interdisciplinary Rehabilitation Research of Greater Montreal
COP: Center of pressure
CS: Cosine similarities
EMG: Electromyography
GM: Gluteus medius
LH: Lateral Hamstring (Biceps femoris)
MH: Medial Hamstring (Semitendinosus)
MG: Medial gastrocnemius
MVE: Maximal voluntary effort
RF: Rectus femoris
RMS: Root mean square
VM: Vastus medialis
SO: Soleus
TA: Tibialis anterior
